# Characterization of novel lignocellulose-degrading enzymes from the porcupine microbiome using synthetic metagenomics

**DOI:** 10.1371/journal.pone.0209221

**Published:** 2019-01-02

**Authors:** Mackenzie Thornbury, Jacob Sicheri, Patrick Slaine, Landon J. Getz, Emma Finlayson-Trick, Jamie Cook, Caroline Guinard, Nicholas Boudreau, David Jakeman, John Rohde, Craig McCormick

**Affiliations:** 1 Department of Microbiology and Immunology, Dalhousie University, Halifax, Nova Scotia, Canada; 2 Department of Chemistry, Dalhousie University, Halifax, Nova Scotia, Canada; 3 College of Pharmacy, Dalhousie University, Halifax, Nova Scotia, Canada; Weizmann Institute of Science, ISRAEL

## Abstract

Plant cell walls are composed of cellulose, hemicellulose, and lignin, collectively known as lignocellulose. Microorganisms degrade lignocellulose to liberate sugars to meet metabolic demands. Using a metagenomic sequencing approach, we previously demonstrated that the microbiome of the North American porcupine (*Erethizon dorsatum*) is replete with genes that could encode lignocellulose-degrading enzymes. Here, we report the identification, synthesis and partial characterization of four novel genes from the porcupine microbiome encoding putative lignocellulose-degrading enzymes: β-glucosidase, α-L-arabinofuranosidase, β-xylosidase, and endo-1,4-β-xylanase. These genes were identified via conserved catalytic domains associated with cellulose- and hemicellulose-degradation. Phylogenetic trees were created for each of these putative enzymes to depict genetic relatedness to known enzymes. Candidate genes were synthesized and cloned into plasmid expression vectors for inducible protein expression and secretion. The putative β-glucosidase fusion protein was efficiently secreted but did not permit *Escherichia coli (E*. *coli)* to use cellobiose as a sole carbon source, nor did the affinity purified enzyme cleave *p*-Nitrophenyl β-D-glucopyranoside (*p*-NPG) substrate *in vitro* over a range of physiological pH levels (pH 5–7). The putative hemicellulose-degrading β-xylosidase and α-L-arabinofuranosidase enzymes also lacked *in vitro* enzyme activity, but the affinity purified endo-1,4-β-xylanase protein cleaved a 6-chloro-4-methylumbelliferyl xylobioside substrate in acidic and neutral conditions, with maximal activity at pH 7. At this optimal pH, *K*_M_, V_max_, and *k*_cat_ were determined to be 32.005 ± 4.72 μM, 1.16x10^-5^ ± 3.55x10^-7^ M/s, and 94.72 s^-1^, respectively. Thus, our pipeline enabled successful identification and characterization of a novel hemicellulose-degrading enzyme from the porcupine microbiome. Progress towards the goal of introducing a complete lignocellulose-degradation pathway into *E*. *coli* will be accelerated by combining synthetic metagenomic approaches with functional metagenomic library screening, which can identify novel enzymes unrelated to those found in available databases.

## Introduction

The production of bio-ethanol from the degradation of lignocellulosic biomass has been proposed as a sustainable solution to the energy crisis [1, 2]. Lignocellulose is made of three carbohydrate polymers; lignin, hemicellulose and cellulose, with lignin being the most abundant [3]. Lignin polymers are covalently linked to the other structural polysaccharides, hindering the ability of carbohydrate acting enzymes (CAZymes) to hydrolyze cellulose and hemicellulose [4]. Current industrial extraction methods include subjecting lignocellulose to high temperatures, high pressures, and strong acids and bases to remove lignin and enable processing of hemicellulose and cellulose polysaccharides [4, 5]. Environmental microbiologists have investigated the microbial communities in landfill leachate [6], forest decomposition layers [7, 8], and in the microbiomes of termites [9] and ruminants [10, 11, 12] to co-opt efficient microbial enzymes for lignocellulose degradation.

The North American Porcupine, *Erethizon dorsatum*, is a hind-gut fermenter with an enlarged cecum packed with microbes that aid digestion of lignified plants, coniferous and deciduous cambium (inner bark), and flowers [13]. For this reason, we characterized the microbiome of the porcupine as an attractive source of enzymes for biomass conversion. Using metagenomic and 16S sequencing approaches with the KEGG database, the undergraduate 2016 Dalhousie international Genetically Engineered Machine (iGEM) team determined that the porcupine microbiome is replete with genes that could encode proteins similar to known lignocellulose-degrading enzymes [14]. This report provided strong genetic evidence for these enzymes but did not provide direct experimental evidence for enzyme activity. To address this knowledge gap, we, the 2017 Dalhousie iGEM team, created a synthetic metagenomic pipeline which allowed us to identify four candidate CAZyme genes involved in cellulose and hemicellulose degradation; β-glucosidase, α-L-arabinofuranosidase, β-xylosidase and endo-1,4-β-xylanase. These putative enzymes were analyzed alongside their 22 closest homologs using phylogenetics to ensure that the catalytic domain was conserved. These genes were synthesized, cloned into expression vectors and affinity-purified; activity of purified enzymes was measured using matched substrates. Among these, we demonstrated that the microbial endo-1,4-β-xylanase could hydrolyse xylobioside linked to a fluorescent molecule at pH 7. To our knowledge, this study is the first to provide functional characterization of a CAZyme from the porcupine microbiome.

## Results

### Identification and cloning of putative microbial enzymes via a synthetic metagenomic pipeline

**Fig 1:**
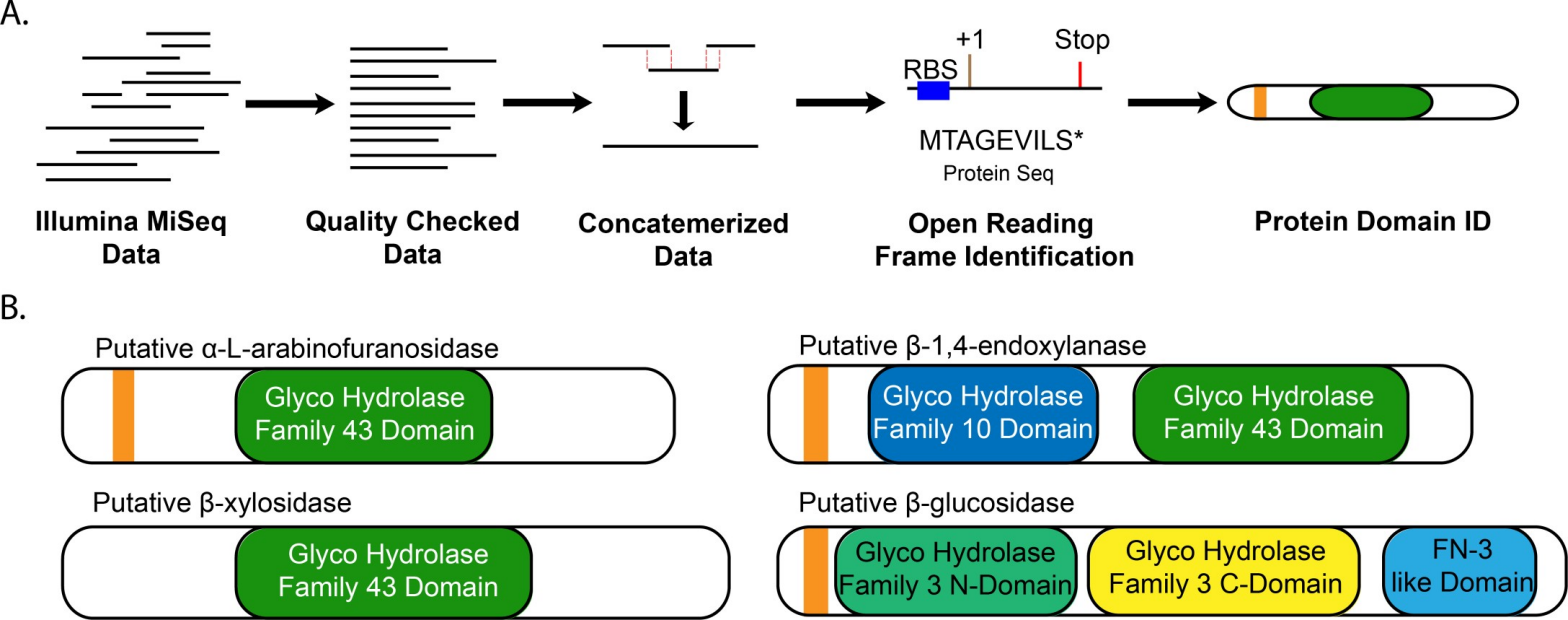
Identification of four genes from the porcupine microbiome with putative cellulose- and/or hemicellulose-degrading activity using a metagenomic sequencing pipeline. The bioinformatic pipeline (A) began with Illumina MiSeq data previously collected from a porcupine fecal DNA sequencing project [14]. Reads were checked for quality and trimmed, concatemerized via MegaHit [15], and open reading frames were identified using Prodigal [16]. Protein sequences of interest were identified by pHMMER [17] using various protein databases and were selected for matches of interest based on e-value selection. (B) Top putative microbial enzymes identified by the metagenomic sequencing pipeline; putative signal sequences are shown in orange and predicted conserved protein domains are shown.

**Fig 2:**
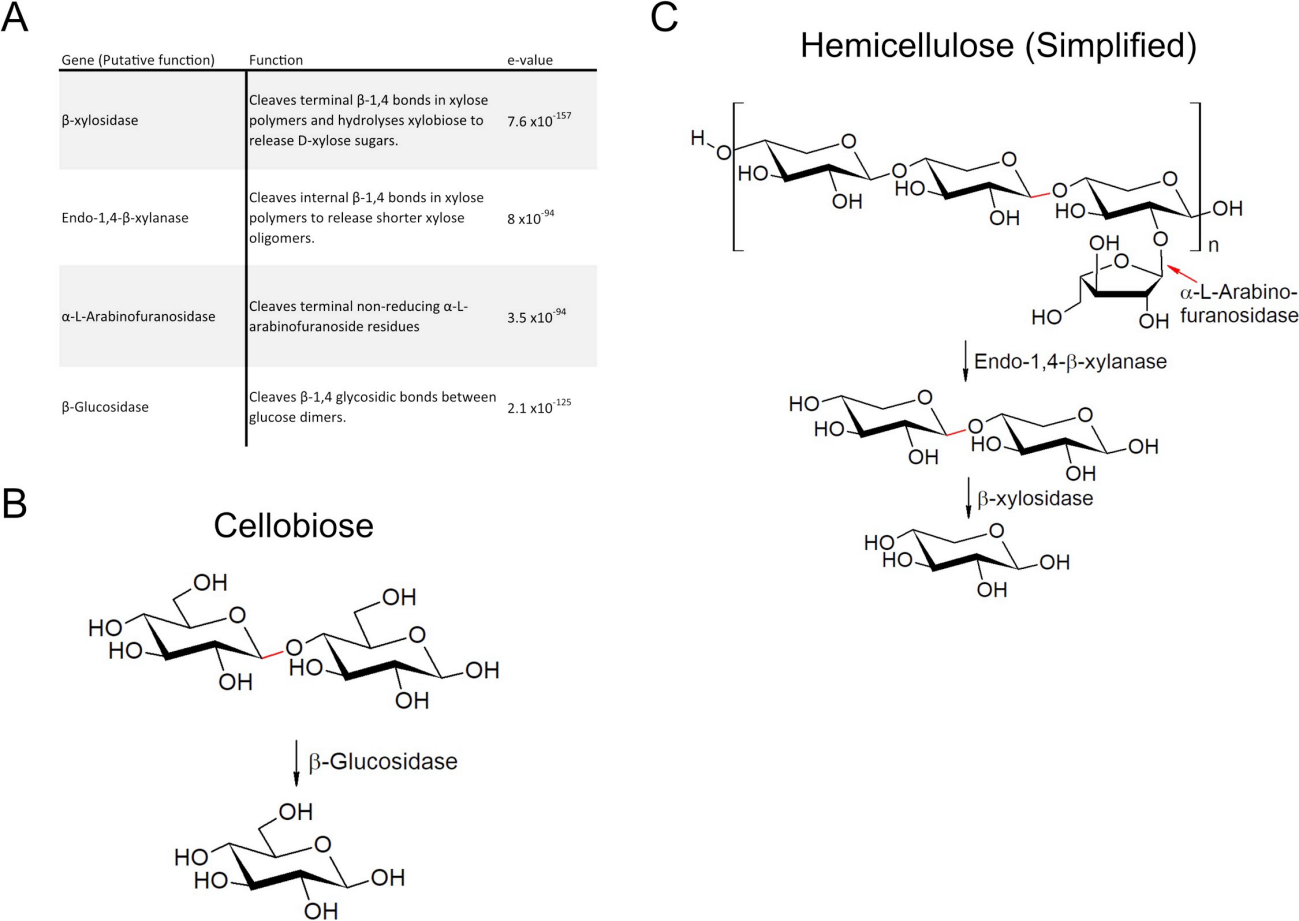
Four putative cellulose-/hemicellulose-degrading enzymes identified from the porcupine microbiome. A) Functional description of putative enzymes with corresponding e-values. The e-value is a measure of confidence, with lower values denoting higher confidence. B) The catabolic pathway that converts cellobiose to glucose. C) The catabolic pathway that converts hemicellulose to xylose.

We used a metagenomic sequencing pipeline (Fig 1A) to identify four microbial genes from porcupine fecal samples predicted to encode putative cellulose- or hemicellulose-degrading enzymes; a β-glucosidase, an α-L-arabinofuranosidase, a β-xylosidase, and an endo-1,4-β-xylanase (Figs 1B and 2A). In brief, shotgun sequences were trimmed and assembled into contigs (Fig 1A). Open reading frames (ORFs) with upstream bacterial ribosome binding sites were identified on the compiled contigs. ORFs were translated *in silico* and identified by similarity of predicted primary amino acid sequences to conserved domains from known enzymes found in the Research Collaboratory for Structural Bioinformatics Protein Data Bank (Fig 1B). We specifically queried β-glucosidase enzymes that catalyze the final step in cellulose degradation by converting cellobiose disaccharides to glucose monomers (Fig 2B), and α-L-arabinofuranosidase, β-xylosidase, and endo-1,4-β-xylanase enzymes that catalyze sequential steps in the degradation of hemicellulose to xylose monomers (Fig 2C). This collection of putative enzymes is insufficient to achieve full degradation of complex lignocellulose substrates, but their synthesis and characterization served as an important test of our synthetic metagenomic pipeline.

### *In silico* analysis of putative enzymes

Metagenomic sequencing sequences all genetic material present in a microbiome rather than individual microbial genomes. To determine the genetic origin of the genes of interest, the predicted primary amino acid sequence of each candidate gene was queried against the NCBI non-redundant protein sequence database using the Basic Local Alignment Search Tool (BLAST) [18]. Each of the four putative enzymes from the porcupine microbiome were most closely related to enzymes encoded by anaerobic bacteria, which was expected because the gut is an anaerobic environment. Specifically, the putative β-xylosidase was 75% identical (100% coverage) to a β-xylosidase from *Butyrivibrio* sp. CAG:318. The putative β-glucosidase was 73% identical (99% coverage) to a β-glucosidase encoded by *Bacteroides faecis* MAJ27. The putative α-L-arabinofuranosidase was 63% identical (92% coverage) to a glycosyl hydrolase (GH) family 43 protein encoded by *Prevotella* sp. CAG:732. The putative endo-1,4-β-xylanase was 55% identical (99% coverage) to a hypothetical protein BHV73_02415 encoded by *Bacteroides* sp. 44_46 and predicted to contain GH10 and GH43 domains. We concluded that the four genes we selected for study were novel but nevertheless closely related to previously identified genes from anaerobic bacteria encoding known cellulose and hemicellulose-degrading enzymes.

**Fig 3:**
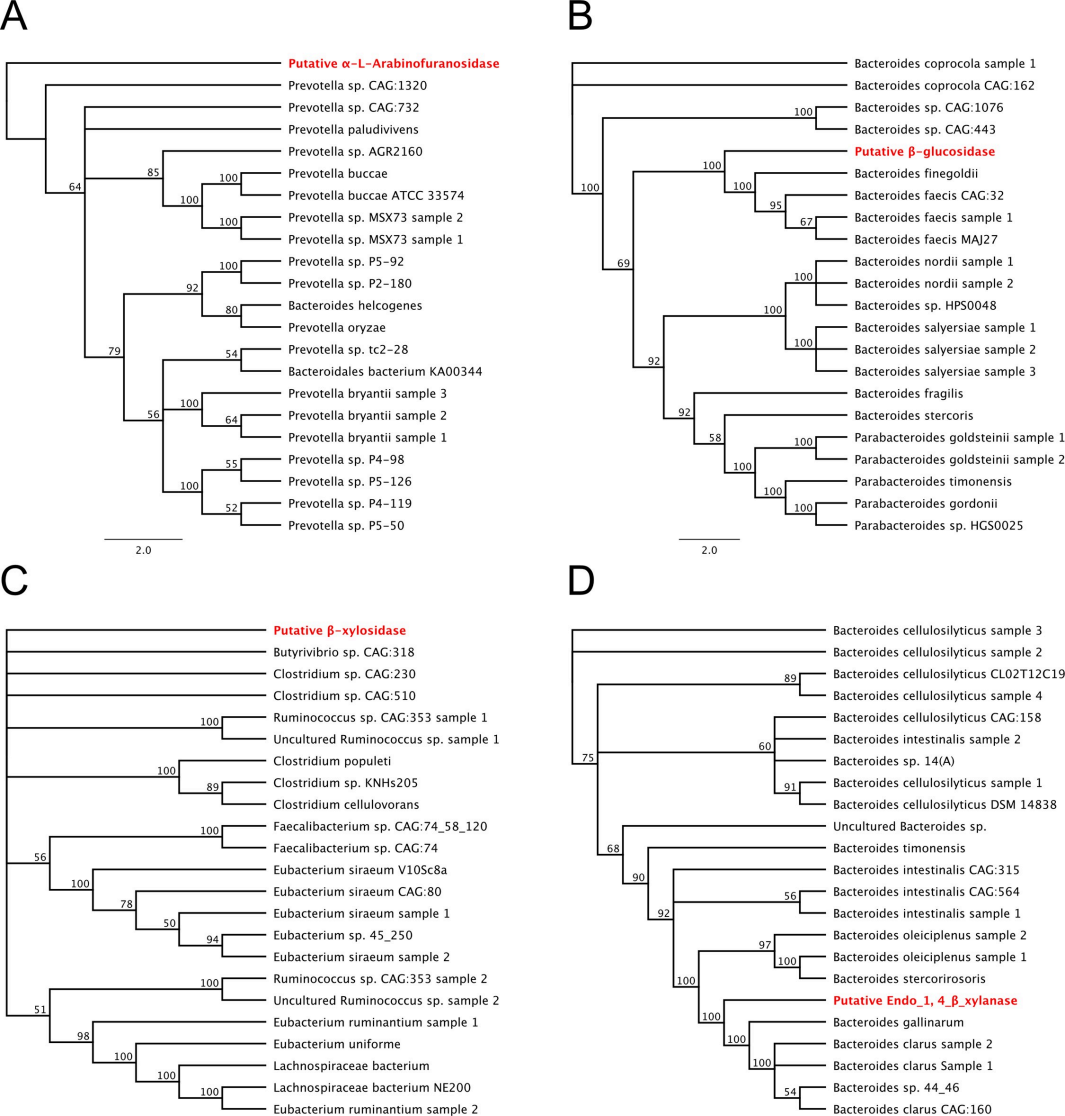
Phylogenetic analysis of putative enzymes. Phylogenetic analysis of A) α-L-arabinofuranosidase, B) β-glucosidase, C) β-xylosidase, and D) Endo-1,4-β-xylanase were created from primary amino acid sequence that were aligned using ClustalW and generated using RAxML 7.2.8. Sequences were taken from highly related proteins from the BLASTP database. Trees were replicated 100 times for statistical strength with bootstrap values presented at each node. Putative enzymes are highlighted in red.

Phylogenetic analysis of all four putative gene products was performed using the 22 closest homologs for each (S1 Table). The putative endo-1,4-β-xylanase and β-glucosidase enzymes clustered closely with their homologs in a clade (Fig 3). These clades were strongly supported by high bootstrap values (100 and 100, respectively) on each node, generated from compiling 100 separate trees. The putative α-L-arabinofuranosidase did not fall into a clade, but did cluster with its closest homologs, whereas β-xylosidase clustered poorly. Next, predicted amino acid sequences of all four putative enzymes were aligned with closest homologs to determine conservation of key catalytic residues (S1A–S1D Fig). A key aspartic acid is conserved in the catalytic domain of the putative β-glucosidase; this residue was conserved across all 22 proteins analyzed, with 4 examples aligned to the putative enzyme in S1A Fig By contrast, the catalytic domain of endo-1,4-β-xylanase consists of two conserved aspartic acids and a glutamic acid; all three residues were perfectly conserved amongst all 22 proteins analyzed and 4 example sequences were aligned to the putative endo-1,4-β-xylanase in S1B Fig α-L-arabinofuranosidase similarly has two conserved aspartic acids and a single glutamic acid in the active site that is perfectly conserved amongst all proteins analyzed (S1B Fig). The putative β-xylosidase showed low conservation from amino acids 190–230, but the conserved catalytic domain requiring the two conserved aspartic acids and a single glutamic acid were also found to be conserved amongst all analyzed sequences. Identifying and confirming the catalytic sites provided evidence that these proteins may function in a biological system and require functional assays to determine activity by *de novo* synthesis of these open reading frames.

### Synthesis, expression and secretion of putative enzymes in *E*. *coli*

**Fig 4:**
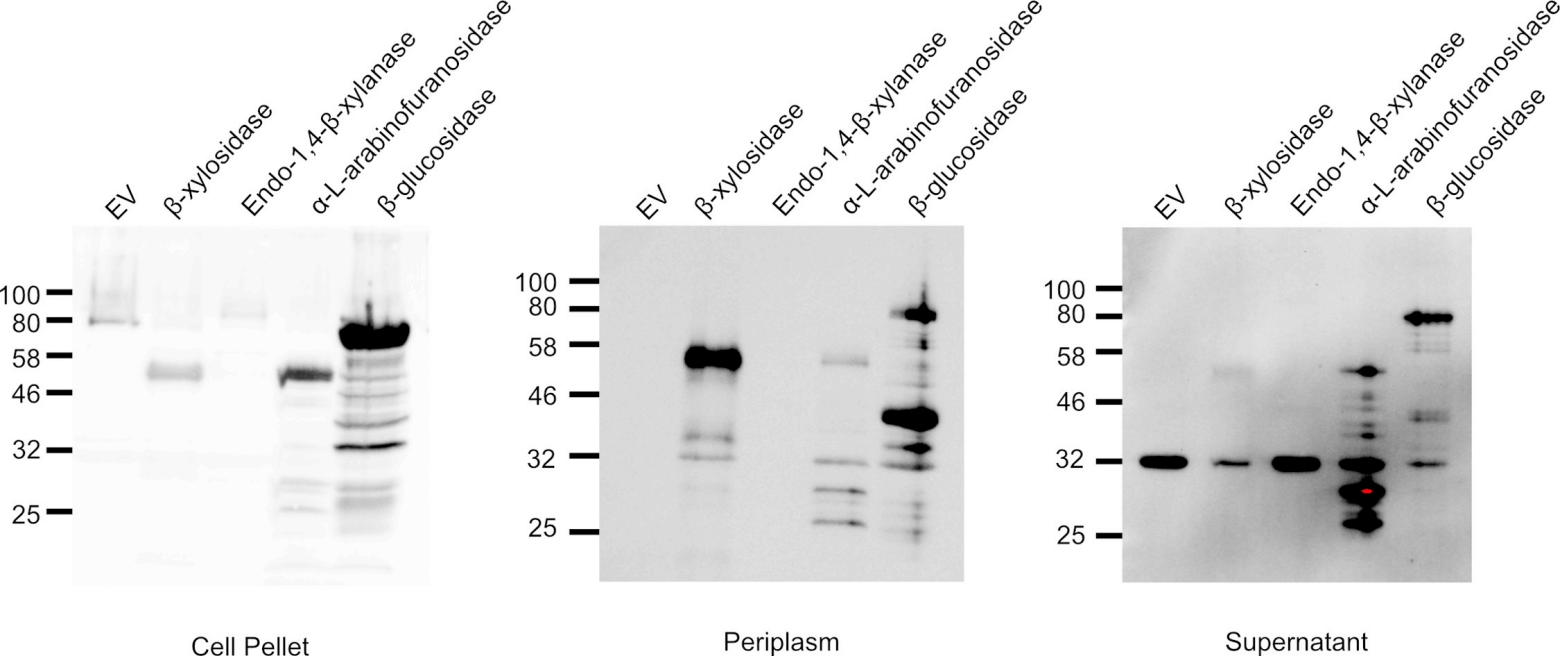
Secretion assay of putative enzymes. Log-phase BL21(DE3) *E*. *coli* containing a putative enzyme or empty vector control were treated with 0.1 mM IPTG to induce T7-promoter-dependent transcription. After 3 hours, A) cells were lysed, B) cells were treated with cold osmotic shock to extract periplasmic protein fraction, or C) the supernatant was filtered, and protein was precipitated by addition of TCA. All protein fractions were prepared for SDS-PAGE and analyzed by western blot with an anti-His-antibody.

The four putative microbial enzymes were synthesized and cloned into the pET26b(+) vector to enable IPTG-inducible gene expression in *E*. *coli*. Because directed secretion of enzymes provides access to extracellular lignocellulosic substrates, each candidate gene was cloned as a fusion protein bearing an amino-terminal PelB motif required for periplasmic localization and subsequent secretion. A hexahistidine (6xHIS) tag was added to carboxy-termini of each fusion protein to enable affinity purification. Log-phase *E*. *coli* cultures were treated with IPTG to induce transgene expression, followed by harvest of cell supernatant, periplasm and total cell fractions. Specifically, proteins in the supernatant were harvested by trichloroacetic acid (TCA) precipitation and periplasmic proteins were harvested by cold osmotic extraction as previously described [19]. These fractions were subjected to SDS-PAGE and immunoblotting to detect 6XHIS fusion proteins (Fig 4). All four putative enzymes accumulated in the total cell fraction at the predicted molecular weight: β-xylosidase at ~51 kDa, endo-1,4-β-xylanase at ~81 kDa, α-L-arabinofuranosidase at ~61 kDa, and β-glucosidase at ~84 kDa (Fig 4A). Among these putative enzymes, only the endo-1,4-β-xylanase failed to translocate to the periplasm, and it accumulated in the cell pellet fraction (Fig 4B and 4C).

### Isolation and characterization of a putative β-glucosidase

**Fig 5:**
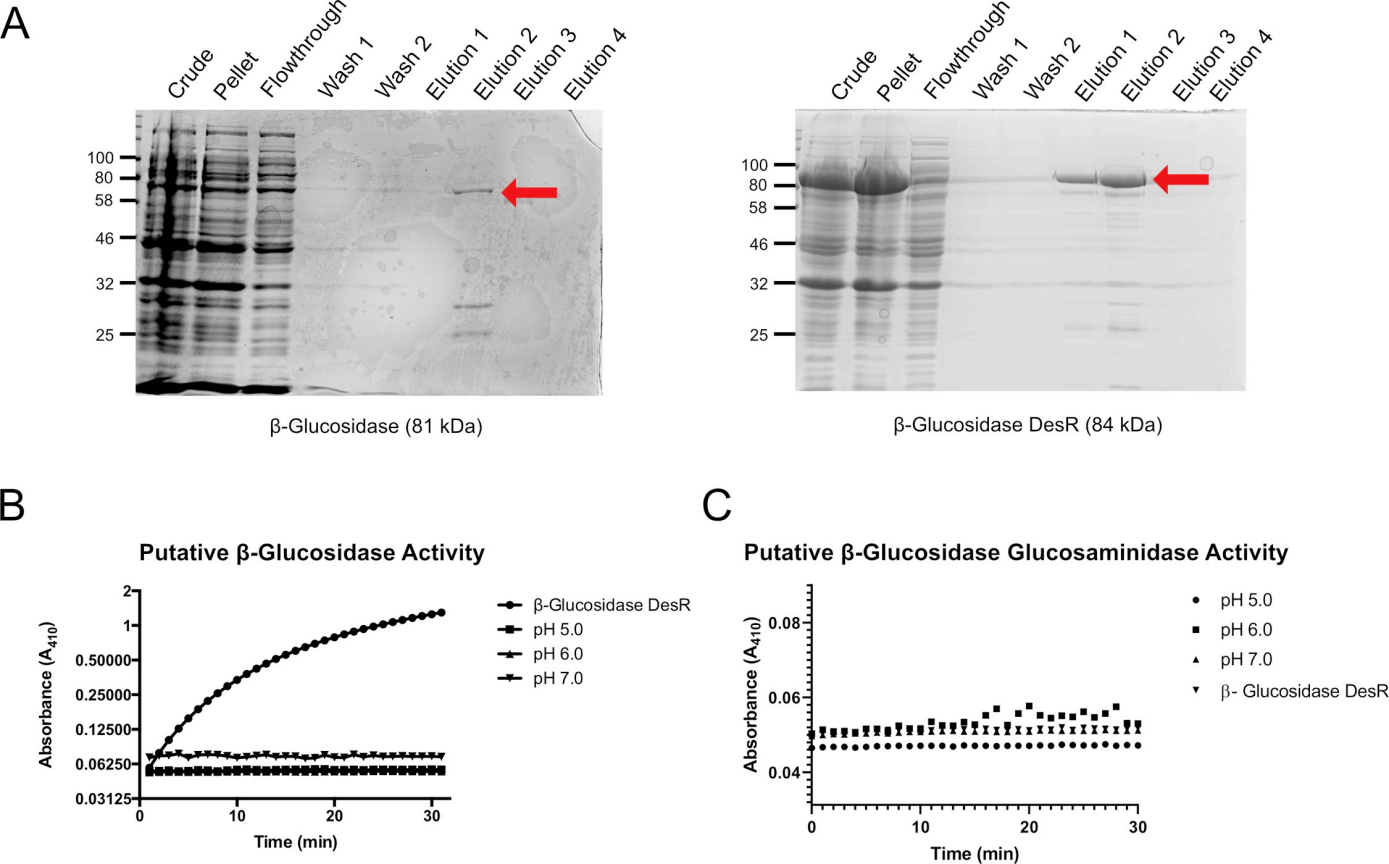
Purification and characterization of β-glucosidase. A) Putative β-glucosidase and positive control β-glucosidase DesR (marked with red arrows) were affinity purified by 6xHis purification. B) Activity of the putative β-glucosidase against *p*-NPG at pH 5.0, 6.0 and 7.0; positive control β-glucosidase DesR was only tested at pH 7.0. C) Activity of putative β-glucosidase against *p-*NP-N-acetylglucosamine at pH 5.0, 6.0, and 7.0. Negative control β-glucosidase DesR was only tested at pH 7.0.

Treatment of transformed log-phase *E*. *coli* cultures with IPTG caused accumulation of our putative microbial β-glucosidase and the control β-glucosidase DesR [20, 21] at predicted molecular weights; both proteins were affinity purified and detected using a total protein stain (Fig 5A). Consistent with published reports, purified DesR efficiently cleaved a *p*-Nitrophenyl β-D-glucopyranoside (*p*-NPG) substrate *in vitro* at pH 7 (Fig 5B). By contrast, our putative β-glucosidase failed to cleave *p*-NPG over a broad pH range. Moreover, expression of the putative β-glucosidase in *E*. *coli* did not enable growth on cellobiose as a sole carbon source. Taken together, these findings indicate that our putative β-glucosidase does not function in conventional β-glucosidase assays. Because proteins with glycosyl hydrolase family 3 (GH3) domains can also have *N*-acetyl-β-D-glucosaminidase activity, we tested our putative β-glucosidase and the DesR control for cleavage of *p*-Nitrophenyl β-*N*-acetylglucosamine (*p*-NPNAG). As expected, DesR did not cleave *p*-NPNAG. However, the putative β-glucosidase also failed to cleave *p*-NPNAG over a broad pH range (pH 5, 6, and 7) (Fig 5C). Taken together, these findings indicate that while the DesR control functioned as expected, the putative β-glucosidase from the porcupine microbiome did not display β-glucosidase or *N*-acetyl-β-D-glucosaminidase activity.

#### Isolation and characterization of putative α-L-Arabinofuranosidase and β-xylosidase enzymes

We purified the putative α-L-arabinofuranosidase using 6xHIS affinity purification as described above. Accumulation of affinity purified 6xHIS-α-L-arabinofuranosidase was detected by Coomassie blue staining (S2A Fig) and immunoblotting with an anti-6XHIS antibody (S2B Fig). Enzyme activity of was assessed using a *p*-NP-α-L-arabinofuranoside (*p*-NP-ALA) substrate. The putative microbial α-L-arabinofuranosidase failed to cleave *p*-NP-ALA over a range of acidic and neutral pH values (5, 6, and 7) (S2C Fig).

Expression of the 6xHIS-β-xylosidase fusion protein revealed that the bulk of the putative enzyme accumulated in the insoluble pellet fraction. To promote proper folding and solubility of our putative β-xylosidase, we reduced IPTG levels from 1.0 mM to 0.1 mM and reduced the culture temperature to 20°C during the time of induction. Despite these efforts, the bulk of the 6xHIS-β-xylosidase fusion protein accumulated in the insoluble pellet, whereas only a small fraction was affinity purified and released into the eluate (S3A Fig). Enzyme activity was tested using *p*NP-β-D-xylopyranoside substrate. A commercial β-xylosidase positive control efficiently cleaved *p*NP-β-D-xylopyranoside over 30 minutes, but the putative microbial β-xylosidase failed to cleave the substrate at pH 5 or pH 6 (S3B Fig). Further testing of this putative microbial β-xylosidase will require mutagenesis to improve protein solubility and increase yield.

#### Isolation and characterization of a putative endo-1,4-β-xylanase

**Fig 6:**
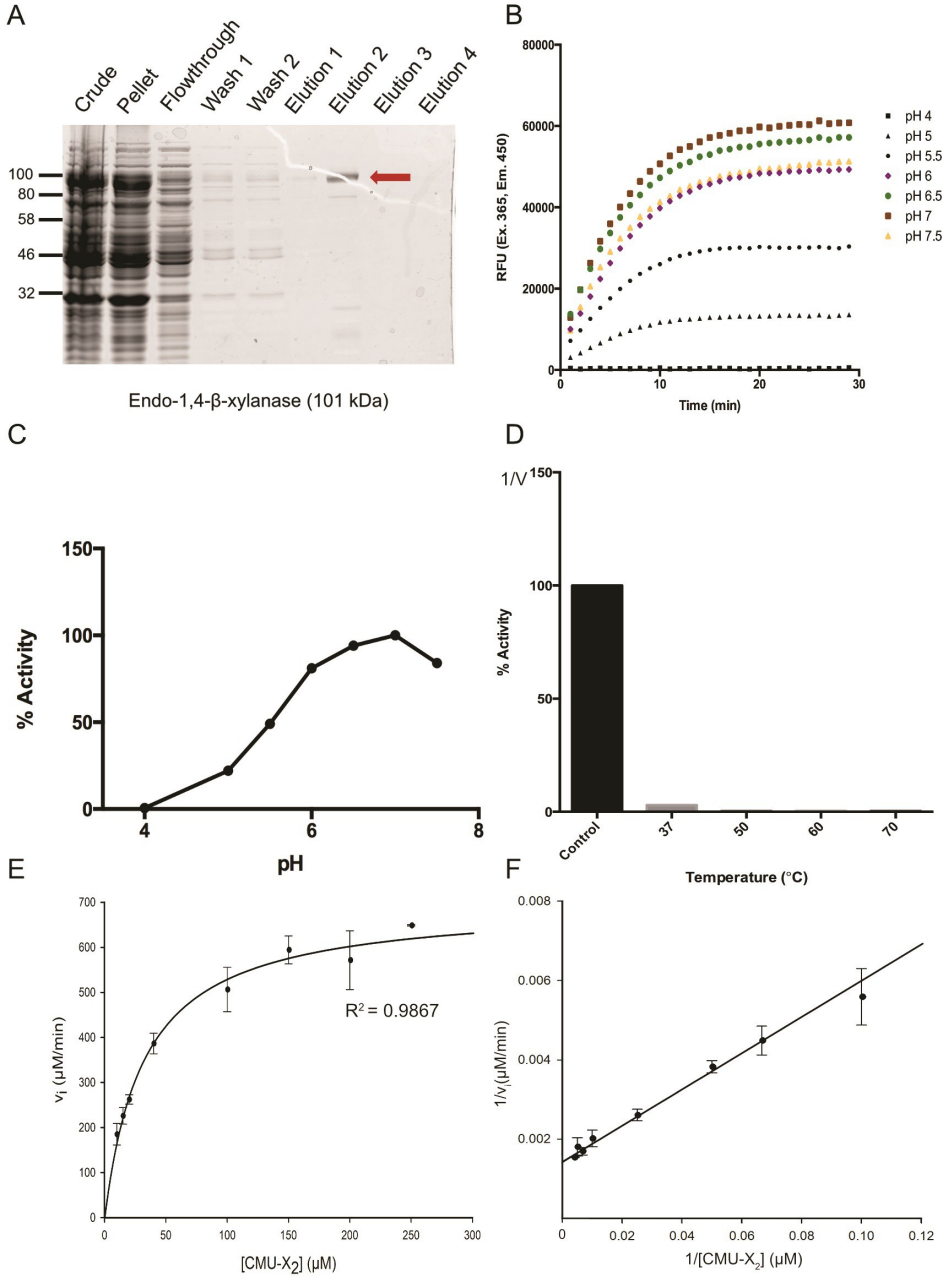
Characterization of endo-1,4- β-xylanase. A) Putative endo-1,4-β-xylanase was purified by 6xHis purification. B) 0.6 μg of endo-1,4-β-xylanase was combined with 100 μM of CMU-X_2_ to assess enzyme activity at different pH values. Samples were assessed via fluorescence in sodium citrate buffer (pH 4, 5, 5.5, 6) or sodium phosphate buffer (pH 6.5, 7, 7.5). A reading of raw fluorescent units (450 nm) was taken every minute for 30 minutes at 37°C. C) Relative activity of endo-1,4-β-xylanase at different pH levels compared to pH 7. D) Thermostability of endo-1,4-β-xylanase was assessed. Samples containing 0.6 μg of endo-1,4-β-xylanase in 50 mM sodium phosphate buffer were incubated for 30 minutes at 37°C, 50°C, 60°C, or 70°C before addition of 100 μM of CMU-X_2_ and fluorescence detection. Control is unincubated endo-1,4-β-xylanase. E) Michaelis-Menten plot was generated with initial reaction rate against substrate concentrations above at 37°C, pH 7. F) Lineweaver-Burk plot was used to generate kinetic constants *K*_M_, V_max_, and *k*_cat_.

We successfully purified the putative endo-1,4-β-xylanase from cell lysates using the protein production and isolation protocol described above (Fig 6A). Enzyme activity was assessed using 6-chloro-4-methylumbelliferyl xylobioside (CMU-X_2_) substrate and a modified protocol developed by Hallam and Withers [22]. When cleaved by an active enzyme, CMU is released from xylobiose, causing fluorescence emission. CMU-X_2_ (100 μM) was combined with 0.6 micrograms of purified putative endo-1,4-β-xylanase across a range of pH values. Immediately upon addition of enzyme, the fluorophore was excited at 365 nm and emission was read at 450 nm; one measurement was taken every minute for 30 minutes. The increase in raw fluorescence units (RFUs) over time reported cleavage of CMU-X_2_ substrate by the putative endo-1,4-β-xylanase; this reaction was catalyzed most efficiently at pH 7 (Fig 6B). The DesR β-glucosidase served as a negative control in these assays and did not cleave the CMU-X_2_ substrate (S4 Fig).

To investigate the thermostability of the putative endo-1,4-β-xylanase, we incubated samples containing 0.6 micrograms of purified enzyme for 30 minutes at 37°C, 50°C, 60°C, or 70°C before conducting an *in vitro* CMU-X_2_ cleavage assay as described above. Enzyme activity was abolished after 30-minute incubation at any of these temperatures (Fig 6D). A Michaelis-Menten plot was generated for endo-1,4-β-xylanase activity over a range of CMU-X_2_ concentrations (Fig 6E). From this, kinetic constants for endo-1,4-β-xylanase were calculated using a Lineweaver-Burk double reciprocal plot (Fig 6F). Endo-1,4-β-xylanase had a calculated *K*_M_ value of 32.005 ± 4.72 μM for CMU-X_2_ and a V_max_ of 1.16x10^-5^ ± 3.55x10^-7^ M/s. The calculated turnover number (*k*_cat_) for endo-1,4-β-xylanase was found to be 94.7 s^-1^. Finally, *k*_cat_/*K*_M_ was calculated to be 2.96x10^6^ M^-1^s^-1^.

## Discussion

The Dalhousie 2016 iGEM team previously demonstrated that the porcupine microbiome is a potentially rich source of lignocellulose-degrading enzymes [14]. We developed a synthetic metagenomic pipeline to mine the porcupine microbiome for novel enzymes with useful properties. Using stringent selection criteria to maximize our chances of discovering *bona fide* cellulose/hemicellulose-degrading enzymes, we identified and synthesized four novel genes encoding putative cellulose- and hemicellulose-degrading enzymes from the porcupine microbiome: a β-glucosidase, an α-L-arabinofuranosidase, a β-xylosidase, and an endo-1,4-β-xylanase. These putative enzymes were thoroughly analyzed by phylogenetics and key conserved residues in proposed catalytic sites were identified. These putative enzymes were affinity purified, and we demonstrated clear *in vitro* activity of a purified putative endo-1,4-β-xylanase, with optimal activity at pH 7, consistent with the neutral pH of the porcupine cecum [23]. This marks the first discovery of a functional hemicellulose-degrading enzyme from the porcupine microbiome, and it demonstrates the power of our synthetic metagenomic approach.

The microbial genome that encodes our novel endo-1,4-β-xylanase is unknown, but this gene has a Shine-Dalgarno (SD) Sequence commonly found in bacterial genes, and phylogenetic analysis revealed that it is most closely related to uncharacterized proteins with glycosyl hydrolase (GH) family 10 and 43 domains from *Bacteroides spp*. as described by the Carbohydrate Active Enzymes Database (CAZy, http://www.cazy.org/) [24]. GH10 domains have been linked to endo-1,4-β-xylanase, endo-1,3-β-xylanase, tomatinase and/or xylan endotransglycosylase activity, while GH43 domains are commonly found in β-xylosidase and/or α-L-arabinofuranosidase enzymes [24]. Because our candidate endo-1,4-β-xylanase cleaved a modified xylobiose substrate *in vitro*, we inferred that the GH10 domain was functional in the context of the affinity-purified fusion protein, despite the addition of an amino-terminal PelB domain and carboxy-terminal 6XHIS affinity purification tag. This enzyme displayed activity across a wide range of pH values (pH 4–7.5) but lacked thermostability, losing activity after 30 min incubation at a range of temperatures as low as 37°C, despite the fact that the porcupine deep core body temperature is approximately 37°C [23, 25]. This thermo-instability makes our novel endo-1,4-β-xylanase less useful for industrial applications compared to known thermophilic endo-1,4-β-xylanases [26]. Enzyme thermostability can be improved via directed evolution; Stephens *et*. *al*. used error-prone PCR to increase endo-1,4-β-xylanase thermostability, creating a superior variant with a single amino acid substitution [27]. Advances in computational prediction and protein folding models can also inform rational mutagenesis strategies to increase enzyme thermostability [28, 29, 30]. Such approaches may be used to enhance the thermostability of our novel endo-1,4-β-xylanase.

We calculated a turnover rate (*k*_cat_) of 94.7 s^-1^ for our novel endo-1,4-β-xylanase, which compares favorably with other known enzymes. Using beechwood xylan as a substrate, He *et*. *al*. reported that an endo-1,4-β-xylanase isolated from the fungus *Trichoderma reesei* had a relatively high *k*_cat_ of 139.7 s^-1^ [8]. Using the same substrate, Xu *et*. *al*. reported a lower rate of turnover of 47.34 s^-1^ for a microbial xylanase containing a GH10 family domain isolated from the feces of the black snub-nosed monkey (*Rhinopithecus bieti*) [31]. A GH11 family endo-1,4-β-xylanase isolated from the fungus *Fusarium oxysporum* also had a low rate of turnover (0.27 s^-1^) of RBB-xylan substrate, a chromogenic derivative of beechwood xylan [32]. Like other kinetic constants, turnover rates are substrate-specific. Because we were the first to use CMU-X_2_ to calculate kinetic constants for an endo-1,4-β-xylanase, we cannot directly compare our findings to studies that employ beechwood xylan or RBB-xylan. Future studies should characterize kinetic constants of our endo-1,4-β-xylanase on more conventional substrates such as beechwood, birchwood, and oat-spelt xylan.

Among the other three putative enzymes identified in this study, the β-glucosidase accumulated to high levels, translocated to the periplasm and was efficiently secreted, yet it lacked *in vitro* activity in *p*-NPG or *p*-NPNAG assays. The putative α-L-arabinofuranosidase was also efficiently secreted but it lacked *in vitro* activity. By contrast, β-xylosidase was difficult to purify and was often retained in the insoluble pellet fraction. Insolubility can occur when a protein aggregates before it can fold properly [33]. To promote proper folding and solubility of our putative β-xylosidase, we reduced IPTG levels from 1.0 mM to 0.1 mM and reduced the culture temperature to 20°C during the time of induction. Despite these precautions, the amount of affinity purified β-xylosidase remained insufficient for subsequent *in vitro* enzyme assays. It is possible that *E*. *coli* lacks appropriate chaperone activity required to efficiently fold this enzyme.

Metagenomic shotgun sequencing allowed us to characterize all genetic material in the porcupine microbiome but did not reveal which organism encoded the gene of interest. However, diverse bacterial populations are known to secrete enzymes into the gut that work cooperatively to degrade lignocellulose [34, 35]. Our goal was to provide a platform to discover these cooperative enzymes from different microbes. Our pipeline allowed the discovery of putative cellulolytic enzymes from the entire microbiome, including unculturable species. We believe that enzyme discovery using our synthetic metagenomic pipeline will provide opportunities to reverse-engineer lignocellulose degradation pathways for industrial applications.

Our synthetic metagenomic pipeline is a powerful tool for discovery, but it infers functional relationships from homology to previously characterized sequences in a database. Thus, the pipeline will likely fail to identify greatly divergent proteins with desirable properties. It may also identify putative enzymes that appear to have conserved functional domains but lack the function predicted by homology. By contrast, functional metagenomic library screens rely on functional assays for gene discovery. Thus, functional metagenomics provides a convenient approach to new gene discovery that nicely complements sequence-based approaches, but with greater potential for discovery of truly novel genes that don’t resemble those in existing databases. Recently, using functional metagenomics, Cheng, *et*. *al*. discovered three novel β-galactosidase enzymes, two of which had conserved domains, and one of which was part of a previously undiscovered enzyme family [36]. To complement our synthetic metagenomics approach, we plan to create and screen a functional metagenomic library from porcupine microbiome DNA to discover novel lignocellulose-degrading enzymes.

## Materials and methods

### Identification of open reading frames

Metagenomic analysis of Illumina MiSeq data was conducted using our previously published protocols [14]. FASTQC and BowTie2 were used to inspect reads for overall quality and to identify contaminants from sequencing. Reads were trimmed to 400 bp in length to remove low-quality terminal sequences from further analysis. MegaHIT alignment software processed reads in FASTq format and stitched reads into longer contigs by identifying overlapping coding regions [15]. Prodigal was used to identify open reading frames (ORFs) by searching sequences in six frames across both DNA strands [16]. A ‘-c’ command modifier in Prodigal was used to ensure the program only detected ORFs with both start and stop codons present. Prodigal was instructed to search for Shine-Dalgarno sequences required for ribosome binding to prokaryotic mRNAs, and non-canonical start codons CUG, GUG and ACG which are typically found in up to 10% of prokaryotic ORFs; these products are often overlooked by conventional searches and absent from many databases [37, 38]. These restrictions were expected to largely limit our hits to prokaryotic genes regulated by Shine-Dalgarno sequences.

### In silico protein function predictions

pHmmer was used to identify putative function of protein domains [17,39]. Protein domains and possible functions were identified using the Research Collaboratory for Structural Bioinformatics Protein Data Bank [40]. E-values were calculated to compare domains identified in putative proteins to known domains in the database [40]. Putative proteins with the lowest e-values were queried against the Basic Local Alignment Search Tool (BLAST) database using pHmmer to identify proteins with major protein domain conservation. Selected candidate genes were codon-optimized for *E*. *coli* and synthesized by Integrated DNA Technologies (IDT, Coralville, IA, USA) as gBlock gene fragments.

### Amino acid sequence analysis

Phylogenetic trees were generated and interpreted in Geneious R 8.1.8. using RAxML version 7.2.8 with the protein model GAMMA LG (algorithm: Rapid bootstrapping with 100 replicate trees for statistical power) [41]. Amino acid alignments were completed using ClustalW alignment with the cost matrix: BLOSUM with a gap open cost of 10 and a gap extend cost of 0.1 [42]. Putative genes were submitted to GenBank with the accession numbers as follows: β-Glucosidase MH590637, α-L-arabinofuranosidase MH590638, β-xylosidase MH590639, Endo-1,4-β-xylanase MH590640.

### Gene cloning

Candidate genes were PCR-amplified from IDT gBlock gene fragments with Phusion High-Fidelity DNA Polymerase according to manufacturer’s instructions (New England Biolabs (NEB), Ipswich, MA, USA). PCR products were purified using the QIAquick gel extraction kit protocol (Qiagen Inc., Toronto, ON, Canada) (Table 1) and cloned into the pET26b(+) expression plasmid (MilliporeSigma); this plasmid enables the creation of fusion proteins with PelB leader sequences required for translocation to the periplasm after which they can be secreted into the extracellular space [43]. Thus, by fusing our putative enzymes to PelB we increased the likelihood of secretion to the extracellular space to access lignocellulosic substrates. Candidate genes and pET26b(+) were digested with restriction endonucleases (NEB) indicated in Table 1 for 1 hour at 37°C. Digested DNA was subjected to agarose gel electrophoresis on a 0.8% agarose gel and purified using the QIAquick gel extraction kit according to manufacturer’s instructions (Qiagen Inc.), then ligated with pET26b(+) plasmid DNA using T4 DNA ligase (NEB). Ligation products were transformed into chemically competent Stbl3 *E*. *coli* via heat-shock transformation [44]. Specifically, 5 μL of ligation products were added to 50 μL of *E*. *coli* suspension in Luria-Bertani (LB) broth, and following heat-shock transformation, 250 μL of LB broth was added during the 1-hour recovery stage and the mixture was subsequently plated on LB agar supplemented with 25 μg/ml kanamycin. Plates were incubated at 37°C for 18–24 hours to allow the growth of transformants. Colonies were picked and inoculated into 5 mL of LB broth, grown overnight to saturation, and plasmid DNA was extracted via QIAprep Spin Miniprep Kit (Qiagen, Inc.). Plasmids were screened by restriction digestion, and processed for Sanger sequencing (Genewiz, South Plainfield, NJ, USA).

**Table 1:** Oligonucleotide primers for PCR amplification of candidate genes.

Candidate Enzyme	Forward Primer	Reverse Primer	Restriction Endonucleases
β-glucosidase	GAATTCAAGGATCCAATGAACAAGACCCAC	AAAAAACTGCAGCATAGCGGCCGCTGAGCTCTTAAGCTT	BamHI/NotI
α-L-arabinofuranosidase	GAATTCTGGATCCAATGGGTCTTGCTATCG	AAAAAACTGCAGCATAGCGGCCGCTGAGCTCTTCTGAG	BamHI/NotI
β-xylosidase	AAAAAAGGATCCTATGGCTAACCCGTACTTACCTG	AAAAAAGAGCTCATTACTTGGTAAACACG AACGCC	BamHI/SacI
endo-1,4-β-xylanase	GAATTCTGGATCCAATGATCAATAAATTACTGTCATCGGCATTGGCA	AAAAAACTGCAGCATAGCGGCCGCTGAGCTCTTAAGCTT	BamHI/NotI

The pET26b(+) vector contains a carboxy-terminal 6xHIS tag downstream from the multiple cloning site (MCS). Initially, the candidate genes were cloned with a translation stop codon that interrupted the 6xHIS tag. Site-directed mutagenesis was used to delete the stop codon, restoring the 6xHIS tag. The constructs were amplified using Phusion High-Fidelity DNA Polymerase according to manufacturer’s instructions (NEB). Fractions of each PCR product were electrophoresed on a 0.8% agarose gel to confirm size. PCR products was purified with QIAquick PCR Purification Kit according to manufacturer’s instructions (Qiagen). After purification, the 6xHIS tagged constructs were ligated using T4 DNA ligase for 1 hr at 37°C (NEB).

### Inducible protein expression

All pET26b(+) 6xHIS tagged candidate genes were transformed into BL21(DE3) *E*. *coli* to enable inducible protein expression. Selected colonies were inoculated into 5 mL of LB broth and incubated at 37°C in a shaker (220 RPM) overnight. The overnight culture was diluted 1:100 in 2.5 mL LB broth and incubated in a shaker until the OD_600_ of the culture reached 0.5–0.8. Once in log phase, 0.1 mM IPTG (Thermo Fisher Scientific, Waltham, MA, USA) was added to induce protein expression. After 3 hours of shaking incubation at 37°C, bacteria were pelleted at 16000 x g for 2 min, supernatant was collected, and pellets were harvested in 2x electrophoresis sample buffer (ESB) or subjected to periplasmic fractionation. Protein was collected from the supernatant as previously described by Sarty *et al*. with some modification [45]. Specifically, 2.5 mL of clarified supernatant was passed through 0.2 μm PEL syringe filter and placed on ice for 10 min before trichloroacetic acid precipitation. The periplasmic fraction was isolated using previously described cold osmotic shock [19]. Briefly, after the bacteria were pelleted, supernatant was removed, and the pellet was resuspended in 625 μL Cell Fractionation Buffer 1 (0.2 M Tris-HCl (pH 8.0), 200 g/L sucrose, 0.1 M EDTA) then incubated at 4°C for 20 min with regular inversion. After incubation, the suspension was centrifuged at 16000 x g for 15 min at 4°C and supernatant was discarded. The pellet was resuspended in 625 μL Cell Fractionation Buffer 2 (0.1 M Tris-HCl (pH 8.0), 0.005 M MgSO_4_, 0.2% SDS, 1% Triton X-100) and incubated for 20 min at 4°C with regular inversion. The suspension was centrifuged as before, and the supernatant was collected as the periplasmic fraction. TCA precipitation was done to concentrate the protein. The vector pET26b(+) (EV) was used as a negative control. Proteins were subjected to SDS-PAGE, transferred to PDVF and immunoblotted using a mouse anti-penta-HIS antibody (Qiagen, Cat. 34660).

### Protein purification

*E*. *coli* bearing pET26b(+)-endo-1,4-β-xylanase, pET26b(+)-β-glucosidase, or pET26b(+)-α-L-arabinofuranosidase plasmids were grown in 5 mL LB broth with 25 μg/mL kanamycin for 6 hrs at 37°C, shaking, from a single clone. After initial incubation, 1 mL of culture was used to inoculate 100 mL of fresh LB broth with 25 μg/mL kanamycin and 1mM IPTG for overnight induction at 30°C, shaking. After induction, the cultures were centrifuged at 3220 x g for 20 min and supernatant was decanted from pellet. The cell pellets were resuspended in Wash Buffer (20 mM Na_2_PO_4_, 500 mM NaCl, pH 8.0) and sonicated for a total of 6 min in 30 sec intervals on ice, then centrifuged at 8000 xg for 30 min at 4°C. Subsequently, supernatant was loaded into a column containing HisPur Cobalt Resin (Thermofisher) pre-equilibrated with Wash Buffer. The column was washed with six volumes of Wash Buffer, followed by elution in Elution Buffer (150 mM imidazole, 20 mM Na_2_PO_4_, 500 mM NaCl, pH 8.0). Glycerol was added to the purified protein at a final concentration of 10% to maintain proper protein folding.

Recombinant strain pET26b(+)-β-xylosidase was difficult to purify and a modified protocol was used as previously described by Zimmermann *et al*. [46]. Briefly, 1L of LB broth (no antibiotic) was inoculated with 5 mL of saturated overnight pET26b(+)-β-xylosidase culture (containing kanamycin). The culture was shaken at 37°C at 220 rpm until the OD_600_ was between 0.4 and 0.7. Once an appropriate optical density was reached, the culture was incubated at 42°C for 10 min, recovered at 37°C for 20 min, placed on ice for 30 min, and recovered again at 37°C for 30 min. Following heat- and cold-shock treatments, the culture was induced with 0.1 mM IPTG and incubated overnight at 20°C while shaking. The following morning, cultures were pelleted and purified as described above.

### *In vitro* enzyme assays

The activity of candidate β-xylosidase, β-glucosidase, and α-L-arabinofuranosidase enzymes was tested using *p*-nitrophenol (pNP) derivatives *p*NP-β-D-xylopyranoside, *p*NP-β-D-glucopyranoside, and *p*NP-α-L-arabinofuranoside, respectively. When cleaved by active enzyme, these compounds release pNP which can be measured by absorbance at 410 nm wavelength. General experimental procedure combined 900 μL of 5 mM pNP derivative in buffer, which was used to blank the spectrophotometer (Nanodrop One, Thermo Scientific), once 100 μL of appropriately diluted enzyme was added, the absorbance was measured at 410 nm every minute for 15 or 30 min. Optimum pH for β-xylosidase was determined using 2 different 50 mM buffers: citrate buffer at pH 5, and phosphate buffer at pH 6. Optimum pH for β-glucosidase was determined by using three different buffers (50 mM) at pH 5: citrate buffer, pH 6: phosphate buffer, and pH 7: phosphate buffer. Optimum pH for α-L-arabinofuranosidase was determined using three different 50 mM buffers: citrate buffer for pH 5 and 5.5, and a phosphate buffer for pH 6. β-glucosidase was assessed for *N*-acetyl-β-D-glucosaminidase activity using *p*NP-N-acetyl-β-D-glucosaminide as described above.

The activity of endo-1,4-β-xylanase was determined using 6-chloro-4-methylumbelliferyl xylobioside (CMU-X_2_). When cleaved by an active enzyme, 6-chloro-4-methylumbelliferyl is released from xylobiose and can be excited to fluoresce at 450 nm. 100 μM CMU-X_2_ was added to 0.6 μg of affinity-purified endo-1,4-β-xylanase in 50 mM sodium citrate buffer (pH 4, 5, 5.5, 6) or sodium phosphate buffer (pH 6.5, 7, 7.5) in a 96-well plate (Grenier). Fluorescence was measured (Excitation 365 nm, Emission 450 nm) at 37°C using a plate reader (Molecular Devices SpectraMax M2) immediately and every minute for 30 minutes to determine optimal pH for enzyme activity. Fluorescence at optimal pH was then compared to a commercial β-glucosidase (DesR) as a negative control as above. Thermostability of the putative endo-1,4-β-xylanase was determined by incubating samples containing 0.6 μg of enzyme for 30 minutes at 37°C, 50°C, 60°C, or 70°C prior to addition of CMU-X_2_. Samples were then read as before. Control is unincubated endo-1,4-β-xylanase. To calculate kinetic constants, initial rate of product formation (cleavage of CMU from xylobiose) was calculated for 0.6 μg of endo-1,4-β-xylanase incubated with 10 μM, 15 μM, 20 μM, 40 μM, 100 μM, 150 μM, 200 μM, and 250 μM CMU-X_2_ substrate. Kinetic constants *K*_M_ and *k*_cat_ for the putative endo-1,4-β-xylanase were determined and represented on a Lineweaver-Burk plot.

### Cellobiose solid-phase growth assay

BL21 *E*. *coli* containing pET26b(+)-β-glucosidase plasmid vector, empty vector negative control, or positive control pET28b(+)-DesR [14] were cultured overnight in 5 mL LB broth with kanamycin (50 μg/ml) at 37°C, shaking. The next day, 1 ml of overnight culture was added to 4 mL of LB broth supplemented with 50 μg/ml kanamycin and 1 mM IPTG and placed in a shaking incubator at 37°C. After 4 hours, 1 ml of culture was plated on cellobiose M9 solid media (33.7 mM Na_2_HPO_4_, 22 mM KH_2_PO_4_, 8.55 mM NaCl, 9.35 mM NH_4_Cl, 12% cellobiose, 1 mM MgSO_4_ and 0.03 mM CaCl_2_) and incubated overnight at 37°C. Colony growth was visually scored on the following day.

## Supporting information

S1 Table NCBI accession numbers for sequences related to candidate genes from porcupine microbiome from NCBI database. (XLSX)

S1 Fig Alignments. Putative S1A) Endo-1,4-β-xylanase, S1B) α-L-arabinofuranosidase, S1C) β-xylosidase, and S1D) β-glucosidase proteins were aligned to 4 related proteins from different bacterial isolates. Alignments were completed using ClustalW and key catalytic site residues are annotated with a (*).
(TIFF)

S2 Fig Purification and enzymatic activity of a putative α-L-arabinofuranosidase. A) Putative α-L-arabinofuranosidase was purified by 6xHIS purification. Total protein expression was assessed. B) Confirmation of expression was determined via immunoblotting using an anti-HIS antibody. C) Activity of α-L-arabinofuranosidase against *p*-NP-ALA at pH 5.0, 5.5, and 6.0.
(TIFF)

S3 Fig Purification of β-xylosidase. A) Putative β-xylosidase was purified by 6xHIS purification. Red arrow indicates relevant band B) Activity of putative β-xylosidase against *p*-NPX at pH 5.0, and, 6.0 and plotted with positive control; commercial β-xylosidase from *Selenomonas rutinantium* (Megazyme, Ireland).
(TIFF)

S4 Fig Enzymatic activity of endo-1,4-β-xylanase versus unrelated β-glucosidase. Enzymatic activity of endo-1,4-β-xylanase was assessed using cleavage of CMU-X_2_ and emission (450 nm) of CMU at 37°C, pH 7, and compared to negative control β-glucosidase DesR.
(TIFF)

## References

[1] Kumar A, Kushal S, Saraf SA, Singh JS. Microbial biofuels: A solution to carbon emissions and energy crisis. Front Biosci-Landmrk. 2018;23: 1789–1802. 10.2741/4673 29772529

[2] Hasunuma T, Okazaki F, Okai N, Hara KY, Ishii J, Kondo A. A review of enzymes and microbes for lignocellulosic biorefinery and the possibility of their application to consolidated bioprocessing technology. Bioresour Technol. 2013;135: 513–22. 10.1016/j.biortech.2012.10.047 23195654

[3] Hongzhang C. Biotechnology of Lignocellulose: Theory and Practice. [Internet]. 1st ed Dordrecht: Springer Netherlands; c2014 http://www.springer.com/gp/book/9789400768970. 10.1007/978-94-007-6898-7

[4] Ravindran R, Jaiswal AK. A comprehensive review on pre-treatment strategy for lignocellulosic food industry waste: Challenges and opportunities. Bioresour Technol. 2016;199: 92–102. 10.1016/j.biortech.2015.07.106 26277268

[5] Wi SG, Cho EJ, Lee DS, Lee SJ, Lee YJ, Bae HJ. Lignocellulose conversion for biofuel: a new pretreatment greatly improves downstream biocatalytic hydrolysis of various lignocellulosic materials. Biotechnol Biofuels. 2015;8(1):228. 26705422 10.1186/s13068-015-0419-4 PMC4690250

[6] Ransom-Jones E, McCarthy AJ, Haldenby S, Doonan J, McDonald JE. Lignoellulose-degrading microbial communities in landfill sites represent a repository of unexplored biomass-degrading diversity. mSphere. 2017;2(4):e00300–17. 10.1128/mSphere.00300-17 28776044 PMC5541161

[7] de Paula RG, Antonieto ACC, Ribeiro LFC, Carraro CB, Nogueira K, Lopes D, et al New genomic approaches to enhance biomass degradation by the industrial fungus *Trichoderma reesei*. Int J Genomics. 2018;2018: 1974151 10.1155/2018/1974151 30345291 PMC6174759

[8] He J, Yu B, Zhang K, Ding X, Chen D. Expression of endo-1, 4-beta-xylanase from Trichoderma reesei in Pichia pastoris and functional characterization of the produced enzyme. BMC Biotechnol. 2009;9(1): 56. 19527524 10.1186/1472-6750-9-56 PMC2702311

[9] Lazuka A, Auer L, O’Donohue M, Hernandez-Raquet G. Anaerobic lignocellulolytic microbial consortium derived from termite gut: Enrichment, lignocellulose degradation and community dynamics. Biotechnol Biofuels. 2018;11:284 10.1186/s13068-018-1282-x 30356893 PMC6191919

[10] Hess M, Sczyrba A, Egan R, Kim TW, Chokhawala H, Schroth G, et al Metagenomic discovery of biomass-degrading genes and genomes from cow rumen. Science. 2011;331(6016): 463–467. 10.1126/science.1200387 21273488

[11] Do TH, Le NG, Dao TK, Nguyen TMP, Le TL, Luu HL, et al J Gen Appl Microbiol. 2018;63(3): 108–116. 10.2323/jgam.2017.08.004 29526926

[12] Gharechai J, Salekdeh GH. A metagenomic analysis of the camel rumen’s microbiome identifies the major microbes responsible for lignocellulose degradation and fermentation. Biotechnol Biofuels. 2018;11: 216 10.1186/s13068-018-1214-9 30083229 PMC6071333

[13] Graham D. Porcupine. South Dakota Department of Game, Fish and Parks, Division of Wildlife. http://www3.northern.edu/natsource/MAMMALS/Porcup1.html.

[14] Finlayson-Trick ECL, Getz LJ, Slaine PD, Thornbury M, Lamoureux E, Cook J, et al Taxonomic differences of gut microbiomes drive cellulolytic enzymatic potential within hind-gut fermenting mammals. PLOS ONE. 2017;12(12): e0189404 10.1371/journal.pone.0189404 29281673 PMC5744928

[15] Li D, Liu CM, Luo R, Sadakane K, Lam TW. MEGAHIT: an ultra-fast single-node solution for large and complex metagenomics assembly via succinct de Bruijn graph. Bioinformatics. 2015;31(10): 1674–6. 10.1093/bioinformatics/btv033 25609793

[16] Hyatt D, Chen WL, LoCascio PF, Miriam L, Larimer FW, Hauser LJ. Prodigal: prokaryotic gene recognition and translation initiation site identification. BMC Bioinformatics. 2010;11: 119 10.1186/1471-2105-11-119 20211023 PMC2848648

[17] Finn RD, Clements J, Eddy SR. (2011). HMMER web server: interactive sequence similarity searching. J Nucleic Acids. 2011;39: W29–W37. 10.1093/nar/gkr367 PMC3125773 21593126

[18] Altschul SF, Gish W, Miller W, Myers EW, Lipman DJ. Basic local alignment search tool. Biochem. Mol. Biol. J. 1990;215(3): 403–10. 10.1016/S0022-2836(05)80360-2 2231712

[19] Neu HC, Heppel LA. The release of enzymes from *Escherichia coli* by osmotic shock and during the formation of spheroplasts. J Biol Chem. 1965;240(9): 3685–3692. 4284300

[20] Zhao L, Beyer NJ, Borisova SA, Lui H. β-Glucosylation as a part of self-resistance mechanism in methymycin/pikromycin producing strain *Streptomyces venezuelae*. Biochemistry. 2003;42: 14794–14804. 10.1021/bi035501m 14674753

[21] Sadeghi-Khomami A, Lumsden MD, Jakeman DL. Glycosidase inhibition by macrolide antibiotics elucidated by STD-NMR spectroscopy. Chem Biol. 2008;15(7): 739–749. 10.1016/j.chembiol.2008.05.017 18635010

[22] Chen HM, Armstrong Z, Hallam SJ, Withers SG. Synthesis and evaluation of a series of 6-chloro-4-methylumbelliferyl glycosides as fluorogenic reagents for screening metagenomic libraries for glycosidase activity. Carbohydr Res. 2016;421: 33–39. 10.1016/j.carres.2015.12.010 26774876

[23] Vispo C, Hume ID. The digestive tract and digestive function in the North American porcupine and beaver. Can J Zool. 1995;73(5): 967–974.

[24] Lombard V, Golaconda Ramulu H, Drula E, Coutinho PM, Henrissat B. The Carbohydrate-active enzymes database (CAZy) 2013. Nucleic Acids Res. 2014;42: D490–D495. 10.1093/nar/gkt1178 24270786 PMC3965031

[25] DeMatteo KE, Harlow HJ. Thermoregulatory responses of the North American porcupine (Erethizon dorsatum bruneri) to decreasing ambient temperature and increasing wind speed. Comp Biochem Physiol B Biochem Mol Biol. 1997;116(3): 339–46. 9114494 10.1016/s0305-0491(96)00256-8

[26] Yeoman CJ, Han Y, Dodd D, Schroeder CM, Mackie RI, Cann IK. Thermostable enzymes as biocatalysts in the biofuel industry. Adv Appl Microbiol. 2010;70: 1–55. 10.1016/S0065-2164(10)70001-0 20359453 PMC4561533

[27] Stephens DE, Rumbold K, Permaul K, Prior BA, Singh S. Directed evolution of the thermostable xylanase from Thermomyces lanuginosus. J Biotechnol. 2007;127(3): 348–54. 10.1016/j.jbiotec.2006.06.015 16893583

[28] Wijma HJ, Floor RJ, Jekel PA, Baker D, Marrink SJ, Janssen DB. Computationally designed libraries for rapid enzyme stabilization. Protein Eng Des Sel. 2014;27(2): 49–58. 10.1093/protein/gzt061 24402331 PMC3893934

[29] Ásgeirsson B, Adalbjörnsson BV, Gylfason GA. Engineered disulfide bonds increase active-site local stability and reduce catalytic activity of a cold-adapted alkaline phosphatase. Biochim Biophys Acta. 2007;1774(6): 679–87. 10.1016/j.bbapap.2007.03.016 17493882

[30] Zhang W, Mullaney EJ, Lei XG. Adopting selected hydrogen bonding and ionic interactions from *Aspergillus fumigatus* phytase structure improves the thermostability of *Aspergillus niger* PhyA phytase. Appl Environ Microbiol. 2007;73(9): 3069–76. 10.1128/AEM.02970-06 17351092 PMC1892878

[31] Xu B, Dai L, Li J, Deng M, Miao H, Zhou J, et al Molecular and biochemical characterization of a novel xylanase from Massilia sp. RBM26 isolated from the feces of *Rhinopithecus bieti*. J Microbiol Biotechnol. 2015;26(1): 9–19. 10.4014/jmb.1504.04021 26387816

[32] Gómez S, Payne AM, Savko M, Fox GC, Shepard WE, Fernandez FJ, et al Structural and functional characterization of a highly stable endo-β-1, 4-xylanase from *Fusarium oxysporum* and its development as an efficient immobilized biocatalyst. Biotechnol Biofuels. 2016;9(1): 191 10.1186/s13068-016-0605-z 27602054 PMC5011838

[33] Marinez-Alonso M, Gonzalez-Montalban N, Garcia-Fruitos E, Villaverde A. Learning about protein solubility from bacterial inclusion bodies. Microb Cell Fact. 2009;8: 4 10.1186/1475-2859-8-4 19133126 PMC2630952

[34] Cortes-Tolalpa L, Salles JF, Dirk van Elsas J. Bacterial synergism in lignocellulose biomass degradation–Complementary roles of degraders as influenced by complexity of the carbon source. Front Microbial. 2017;8: 1628. 10.3389/fmicb.2017.01628 PMC5641323 29067002

[35] Busk PK, Lange M, Pilgaard B, Lange L. Several genes encoding enzymes with the same activity are necessary for aerobic fungal degradation of cellulose in nature. PLoS One. 2014;9(12): e114138 10.1371/journal.pone.0114138 25461894 PMC4252092

[36] Cheng J, Romantsov T, Engel K, Doxey AC, Rose DR, Neufield JD, et al Functional metagenomics reveals novel β-galactosidases not predictable from gene sequences. PLoS ONE. 2017;12(3): e0172545 10.1371/journal.pone.0172545 28273103 PMC5342196

[37] Kozak M. Initiation of translation in prokaryotes and eukaryotes. Gene. 1999;234(2): 187–208. 10395892 10.1016/s0378-1119(99)00210-3

[38] Hecht A, Glasgow J, Jaschke PR, Bawazer LA, Munson MS, Cochran JR, et al Measurements of translation initiation from all 64 codons in E. coli. Nucleic Acids Res. 2017;45(7): 3615–3626. 10.1093/nar/gkx070 28334756 PMC5397182

[39] Johnson SL, Eddy SR, Portugaly E. Hidden Markov model speed heuristic and iterative HMM search procedure. BMC Bioinformatics. 2010;11: 431 10.1186/1471-2105-11-431 20718988 PMC2931519

[40] Berman HM, Westbrook J, Feng Z, Gilliland G, Bhat TN, Weissig H, et al The Protein Data Bank Nucleic Acids Research. 2000;28: 235–242. 10592235 10.1093/nar/28.1.235 PMC102472

[41] Kearse M, Moir R, Wilson A, Stones-Havas S, Cheung M, Sturrock S, et al Geneious Basic: An integrated and extendable desktop software platform for the organization and analysis of sequence data. Bioinformatics. 2012;28: 1647–1649. 10.1093/bioinformatics/bts199 22543367 PMC3371832

[42] Larkin MA, Blackshields G, Brown NP, Chenna R, McGettigan PA, McWilliam H, et al Clustal W and Clustal X version 2.0. Bioinformatics. 2007;23(21): 2947–2948. 10.1093/bioinformatics/btm404 17846036

[43] Sockolosky JT, Szoka FC. Periplasmic production via the pET expression system of soluble, bioactive human growth hormone. Protein Expr Purif. 2013;87(2): 129–135. 10.1016/j.pep.2012.11.002 23168094 PMC3537859

[44] Froger A, Hall JE. Transformation of plasmid DNA into E. coli using the heat shock method. J Vis Exp. 2007;1(6): 253. 10.3791/253 PMC2557105 18997900

[45] Sarty D, Baker NT, Thomson EL, Rafuse C, Ebanks RO, Graham LL, et al Characterization of the type III secretion associated low calcium response genes of *Vibrio parahaemolyticus* RIMD2210633. Can J Microbiol. 2012;58: 1306–1315. 10.1139/w2012-109 23145828

[46] Zimmermann S, Hall L, Riley S, Sørenson J, Amaro RE, Schnaufer A. A novel high-throughput activity assay for the trypanosoma brucei editosome enzyme REL1 and other RNA ligases. Nucleic Acids Res. 2016 44(3): e24 10.1093/nar/gkv938 26400159 PMC4756849

