# Supplementary figures and images for: Characterization of novel lignocellulose-degrading enzymes from the porcupine microbiome using synthetic metagenomics
Source: PLoS One. 2019 Jan 2;14(1):e0209221. doi: 10.1371/journal.pone.0209221 (PMC6314593; doi:10.1371/journal.pone.0209221)

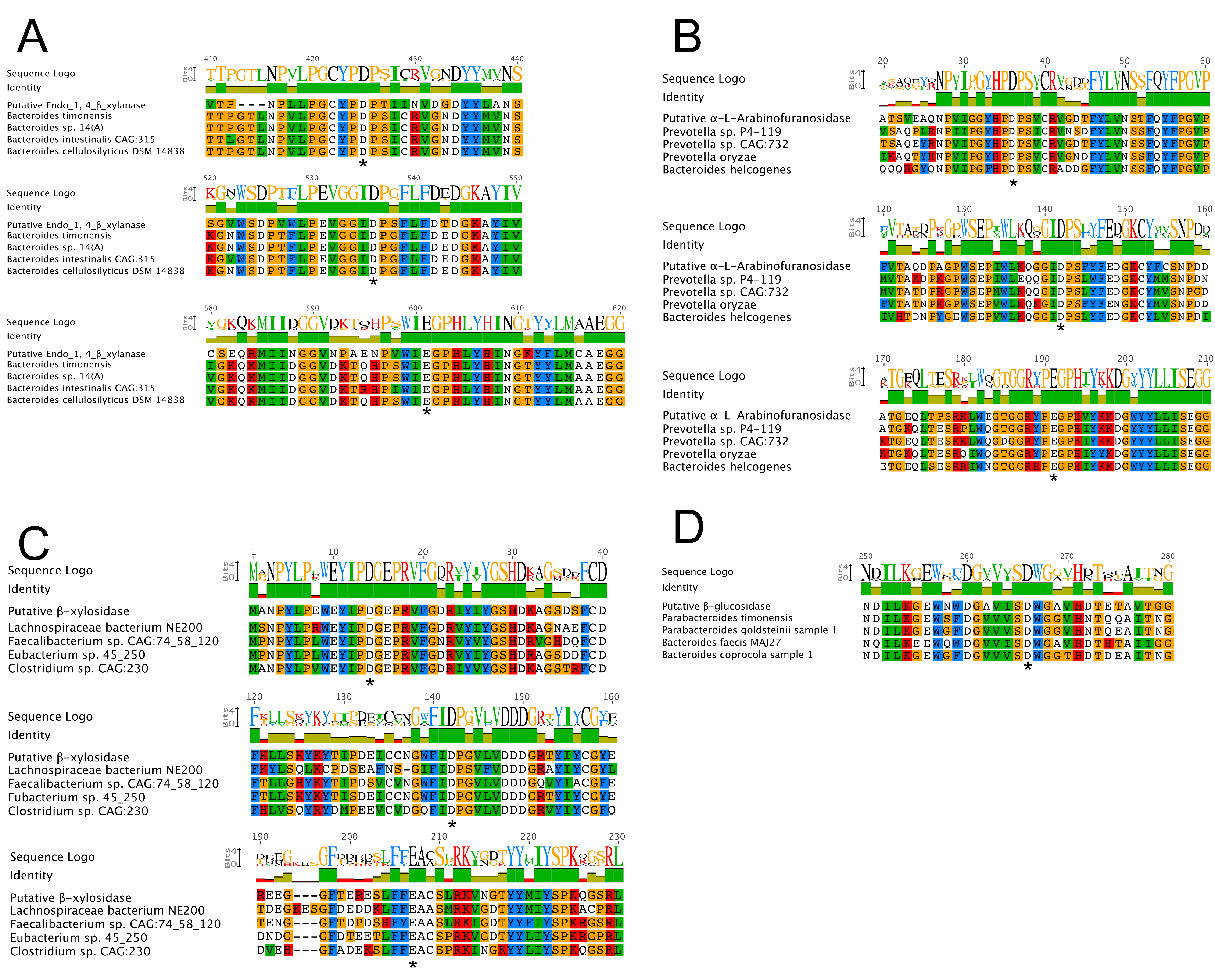

Supplement: S1 Fig — Putative S1A) Endo-1,4-β-xylanase, S1B) α-L-arabinofuranosidase, S1C) β-xylosidase, and S1D) β-glucosidase proteins were aligned to 4 related proteins from different bacterial isolates. Alignments were completed using ClustalW and key catalytic site residues are annotated with a (*). (TIFF) [file pone.0209221.s002.tiff]

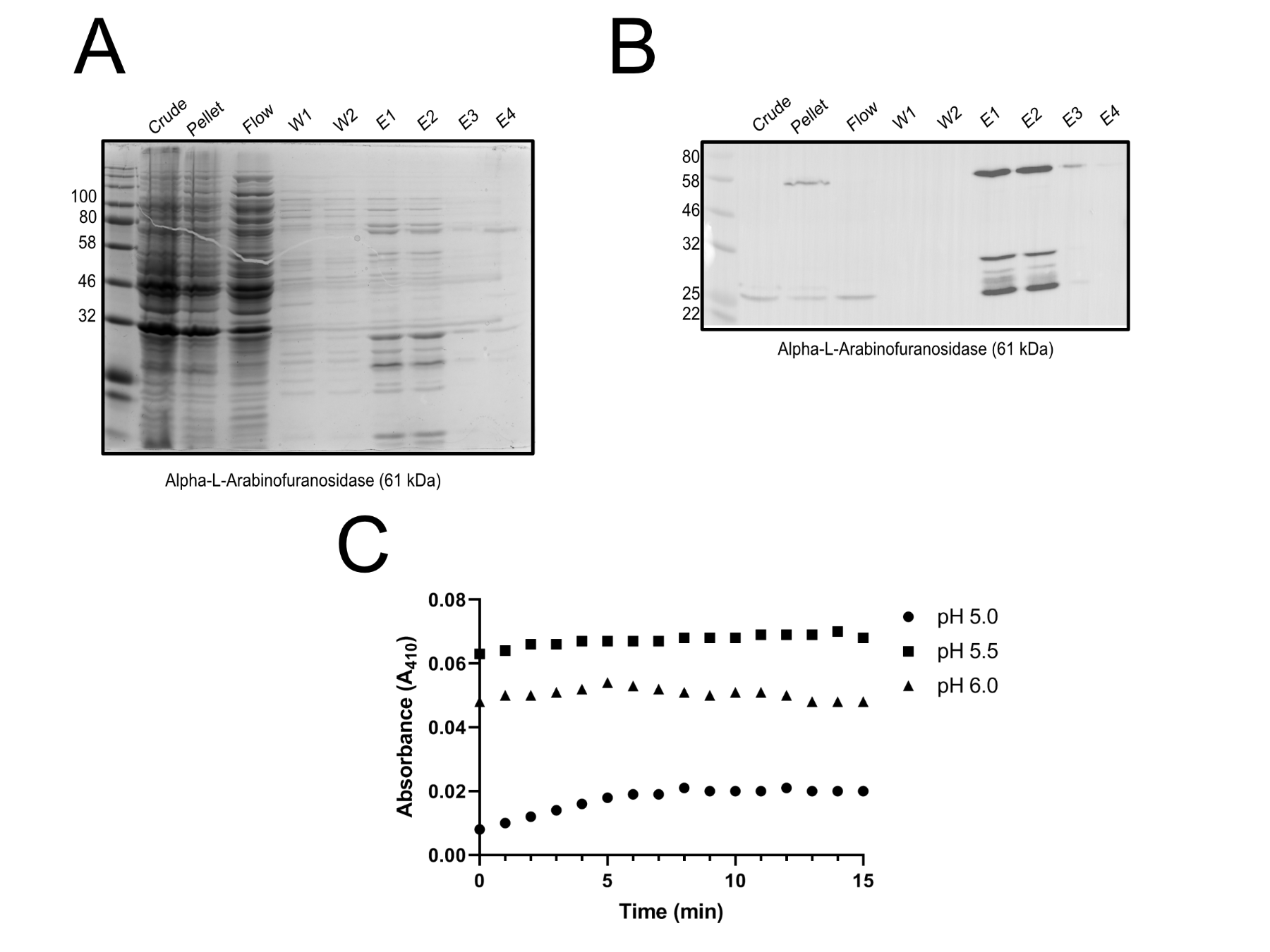

Supplement: S2 Fig — A) Putative α-L-arabinofuranosidase was purified by 6xHIS purification. Total protein expression was assessed. B) Confirmation of expression was determined via immunoblotting using an anti-HIS antibody. C) Activity of α-L-arabinofuranosidase against p-NP-ALA at pH 5.0, 5.5, and 6.0. (TIFF) [file pone.0209221.s003.tiff]

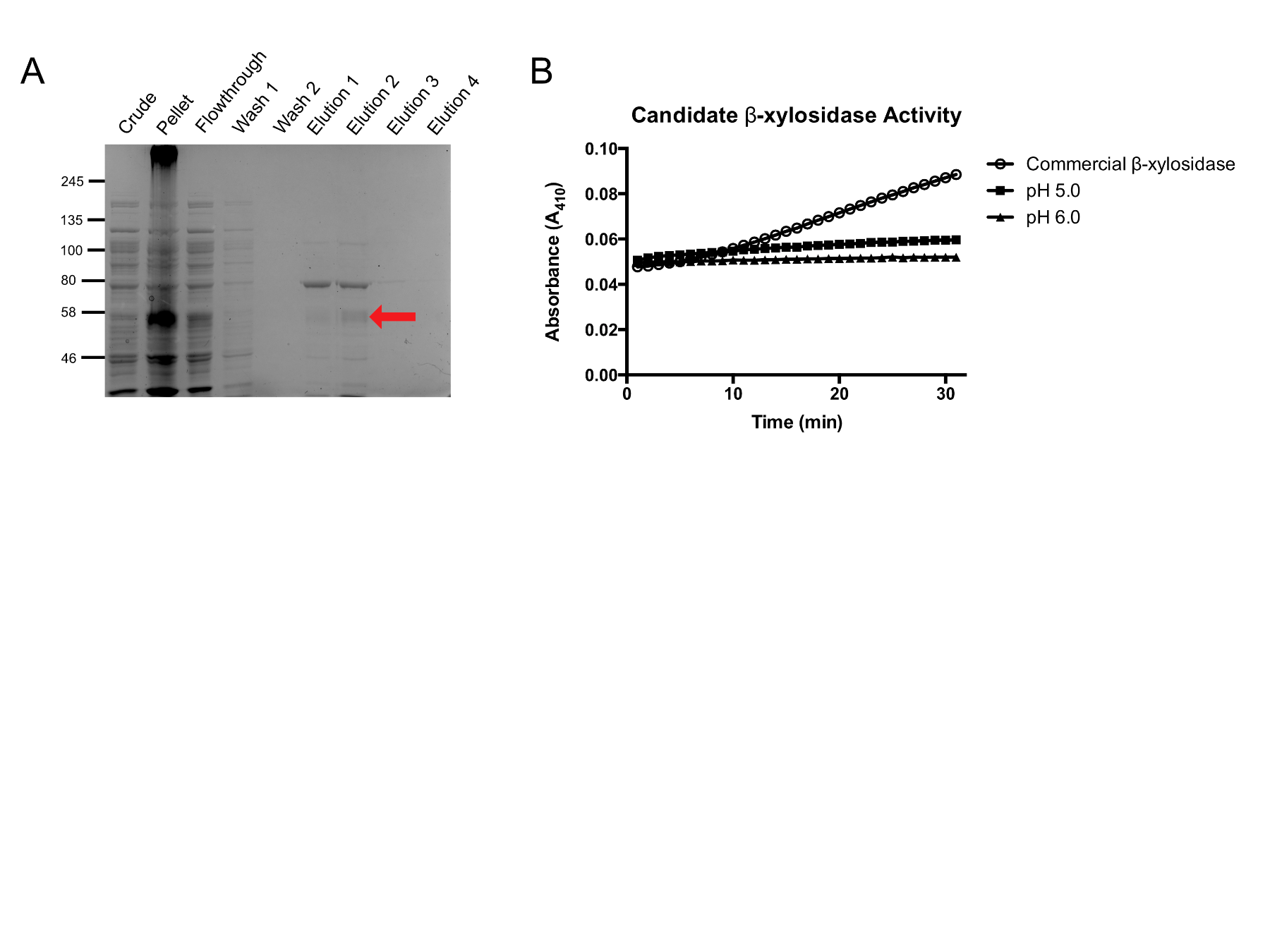

Supplement: S3 Fig — A) Putative β-xylosidase was purified by 6xHIS purification. Red arrow indicates relevant band B) Activity of putative β-xylosidase against p-NPX at pH 5.0, and, 6.0 and plotted with positive control; commercial β-xylosidase from Selenomonas rutinantium (Megazyme, Ireland). (TIFF) [file pone.0209221.s004.tiff]

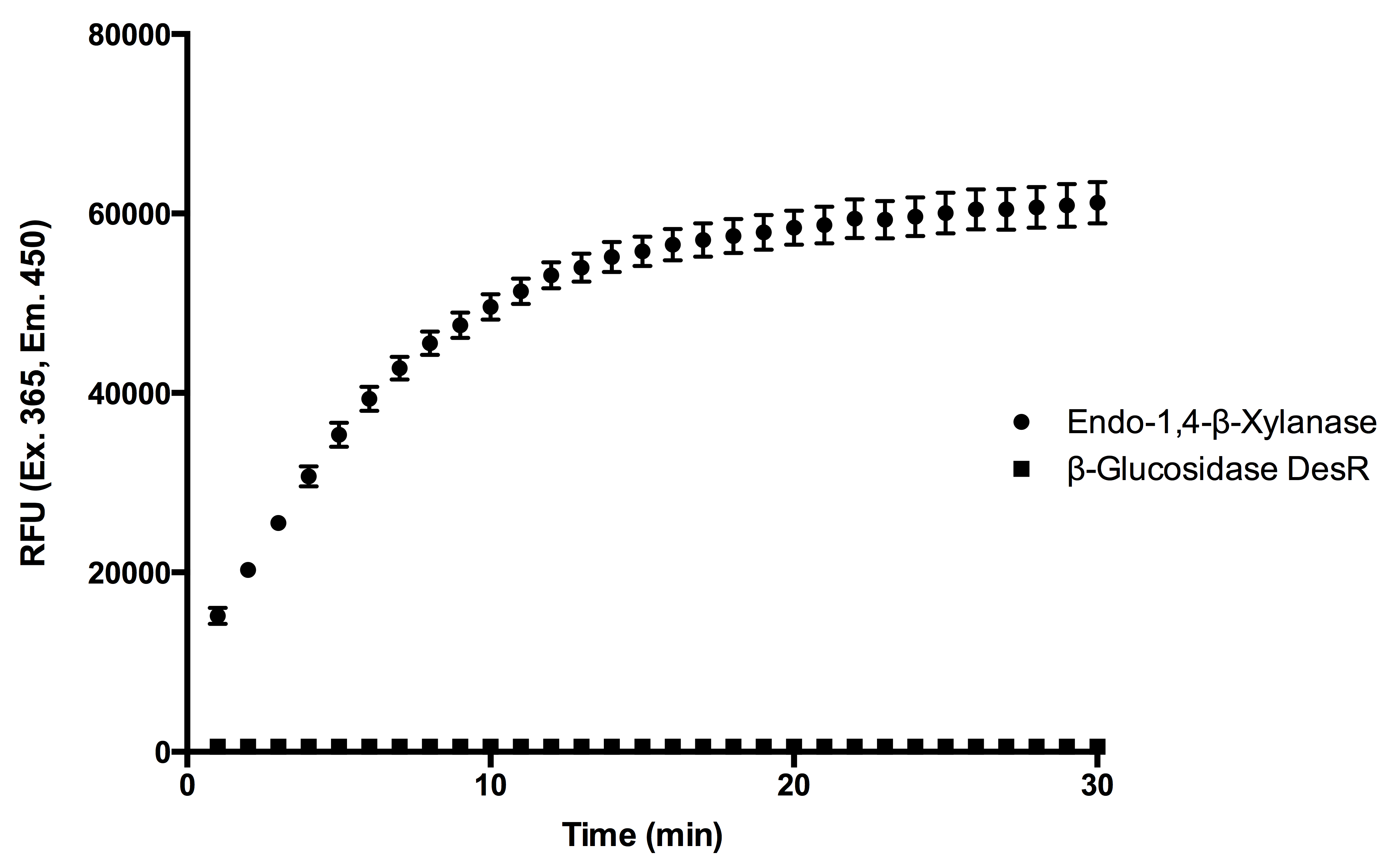

Supplement: S4 Fig — Enzymatic activity of endo-1,4-β-xylanase was assessed using cleavage of CMU-X2 and emission (450 nm) of CMU at 37°C, pH 7, and compared to negative control β-glucosidase DesR. (TIFF) [file pone.0209221.s005.tiff]
